# Prosthetic Rehabilitation of Post-traumatic Eye Defect With a Custom Ocular Prosthesis: A Case Report

**DOI:** 10.7759/cureus.111271

**Published:** 2026-06-22

**Authors:** Akhila Donthoji, Sreeramulu Basapogu, Arifa Mahmood

**Affiliations:** 1 Prosthodontics, Government Dental College and Hospital, Hyderabad, IND

**Keywords:** customised ocular prosthesis, esthetics, eye defect, prosthetic rehabilitation, trauma

## Abstract

Loss of an eye can have significant psychological and social effects on a patient’s well-being. It may result from trauma, infection, congenital conditions, or maxillofacial malignancy. Rehabilitation of an ocular defect with a prosthesis can restore facial harmony and a natural appearance in anophthalmia patients. It also helps improve confidence, esthetics, self-esteem, and quality of life. Stock-eye prostheses are commonly used for the rehabilitation of ocular defects. However, they may not provide an accurate fit due to size mismatch and poor adaptation to the tissue bed, which can compromise eye movement and esthetic outcomes. This case report describes a simple, cost- and time-effective method for fabricating a custom-made acrylic ocular prosthesis for a patient who underwent evisceration of the left eye following a road traffic accident. A customized prosthesis offers better retention and esthetics than a stock eye and can be used successfully in the rehabilitation of patients with ocular defects.

With the development of novel materials and techniques, esthetic expectations for maxillofacial prostheses have increased. Patient-specific customization of an ocular prosthesis can achieve superior aesthetic outcomes.

## Introduction

Eyes, often referred to as the “window to the soul,” are a vital sensory organ enabling vision and are necessary for coordinated movement and stability. Loss of an eye can have a profound psychological impact on a patient’s well-being and self-esteem. Trauma is the most significant cause of loss of an eye, followed by infections, congenital anomalies, and intraocular malignancies [1].

Enucleation or evisceration causes constriction of the tissues around the orbit. The rehabilitation with a prosthesis helps limit this tissue contraction. Fabrication of a definitive ocular prosthesis must follow adequate tissue healing. It ensures defect integrity and restores esthetics, further enhancing the quality of life of the patient [2].

Ocular prostheses have evolved from early designs made of noble metals such as gold and silver to modern materials such as plastics and glass [1]. Methyl methacrylate resin is a biocompatible, aesthetic, durable, and cost-effective material for fabricating ocular prostheses [3]. William Daniel Barker is credited with fabricating the first Polymethyl Methacrylate (PMMA) prosthetic eye in 1942 for his son, who had lost an eye in an accident [1].

The fabrication of a custom ocular prosthesis is a meticulous process that requires both artistic skill and clinical expertise [1]. It involves customizing the scleral shell to fit the patient’s tissue bed, followed by iris color matching and hand painting.

This case report describes the fabrication of a customized ocular prosthesis made of acrylic.

## Case presentation

Initial examination and treatment planning

**Figure 1:**
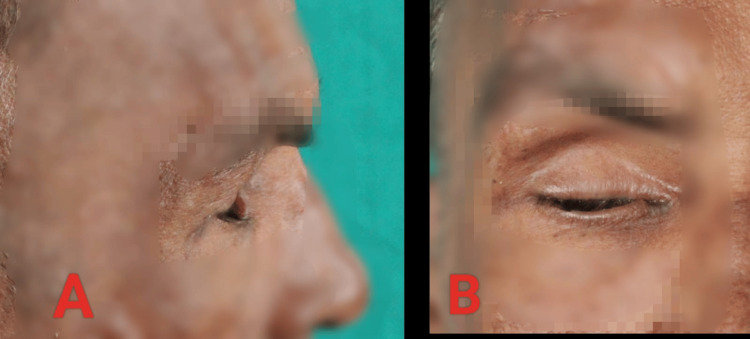
Preoperative images of the patient. A: Right lateral. B: Frontal

A 50-year-old male patient reported to the Department of Prosthodontics. The patient had sustained traumatic injury to the right eye following a road traffic accident around six months earlier. The evisceration of the right eye was performed at the time (Figure 1). Now, the patient wished for an aesthetic rehabilitation of the ocular defect.

Clinical examination of the eye defect revealed adequate tissue bed healing, depth, and mobility between the upper and lower eyelids. The patient did not complain of any pain or tenderness on palpation. A custom acrylic ocular prosthesis for the right eye was planned upon discussion with the patient.

Procedure

**Figure 2:**
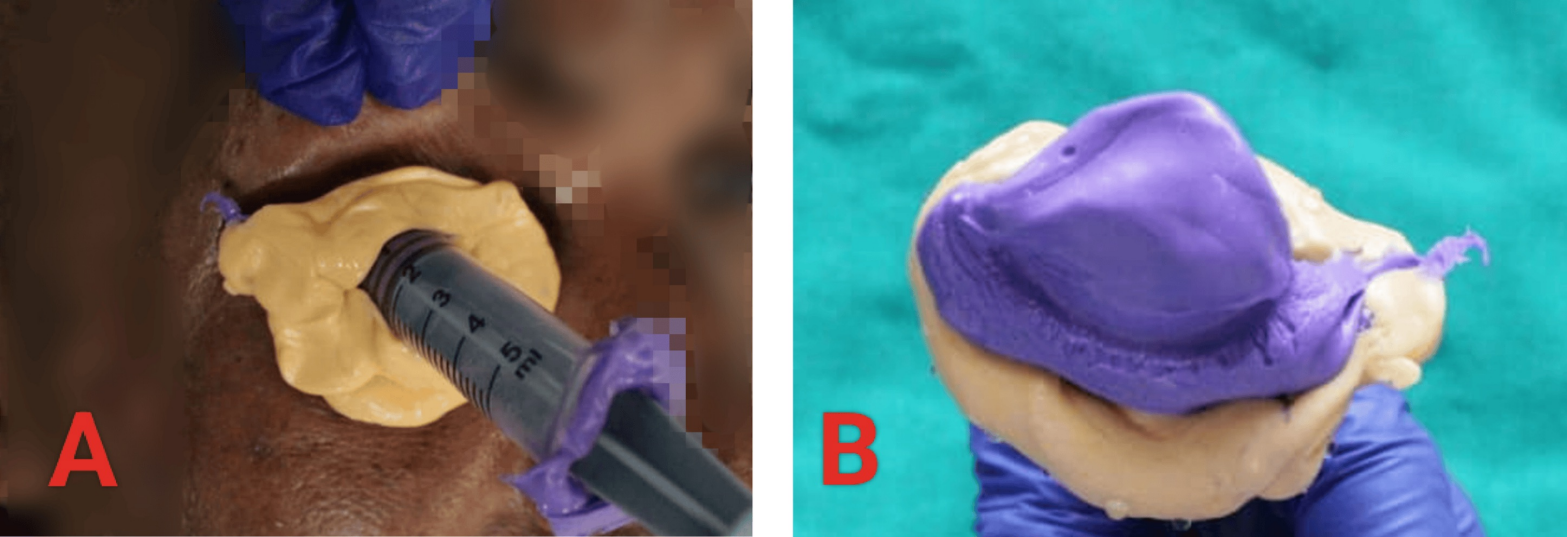
Preliminary impression. A: Adaptation of material. B: Intraocular primary impression

**Figure 3:**
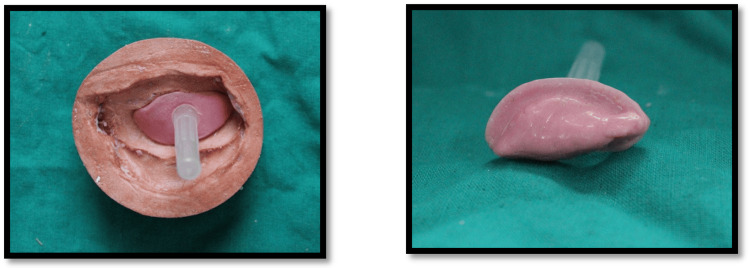
Custom acrylic tray

**Figure 4:**
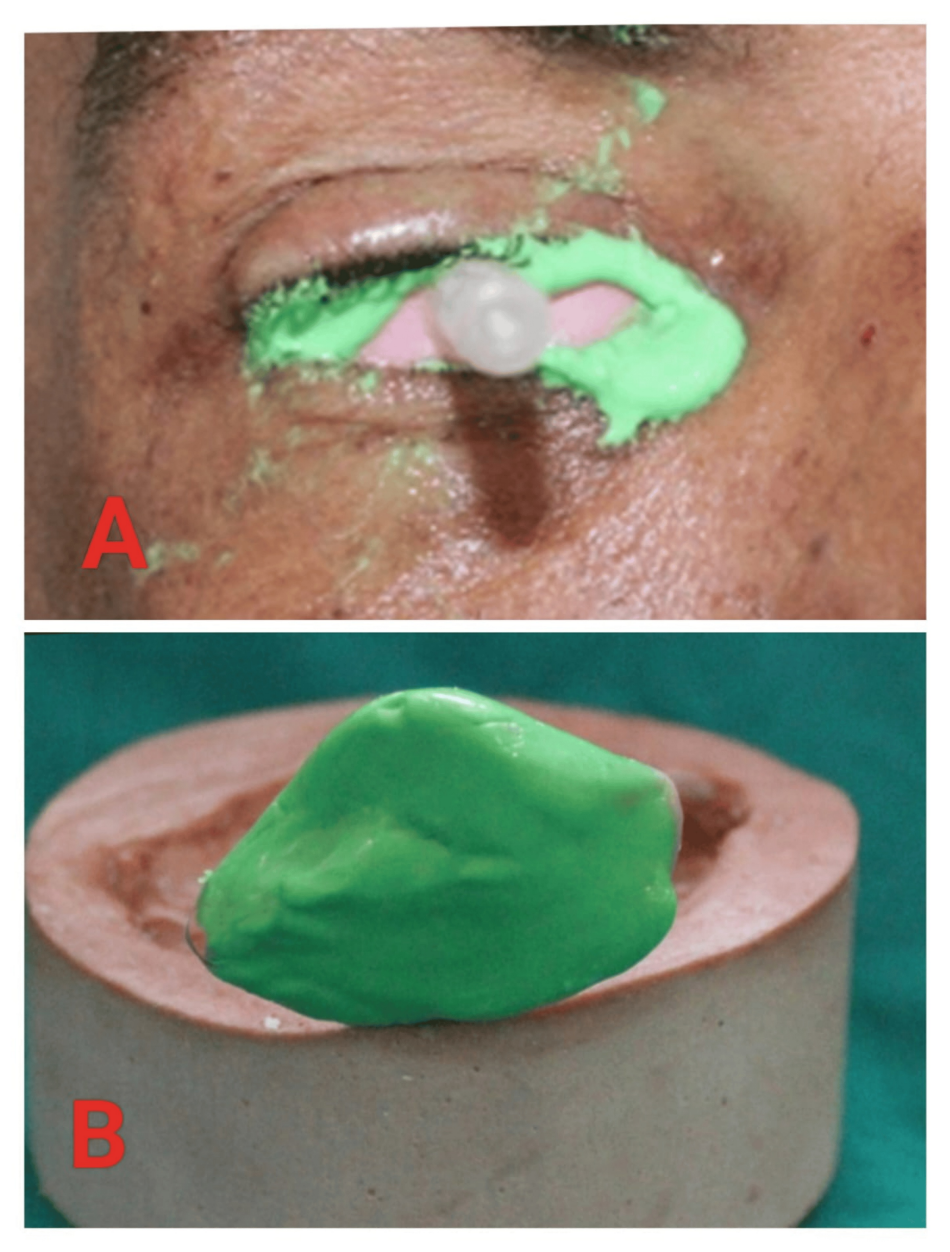
A: Intraocular final impression. B: Final impression made with light body polyvinyl siloxane material

**Figure 5:**
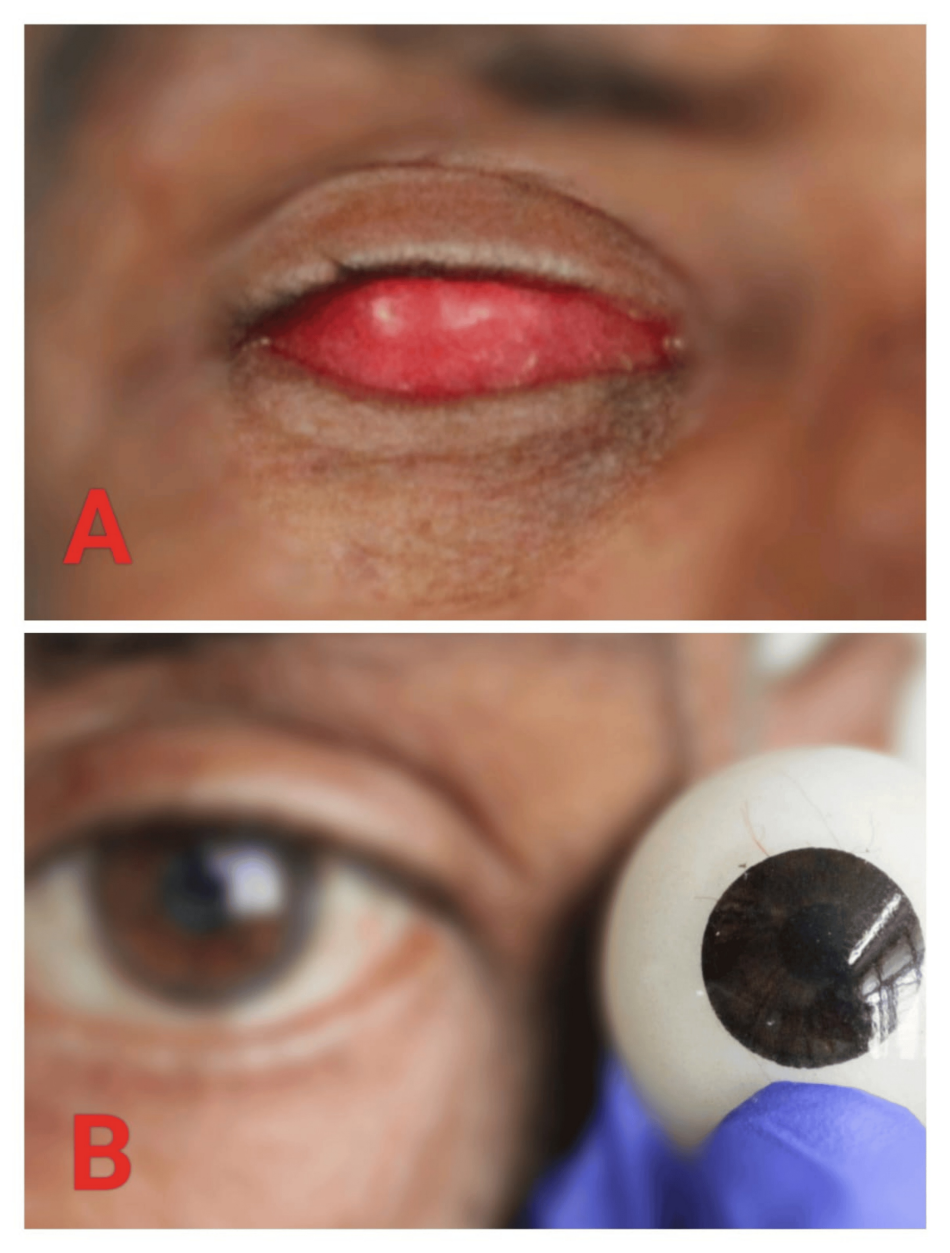
A: Wax-pattern try-in. B: Shade matching using stock eye

**Figure 6:**
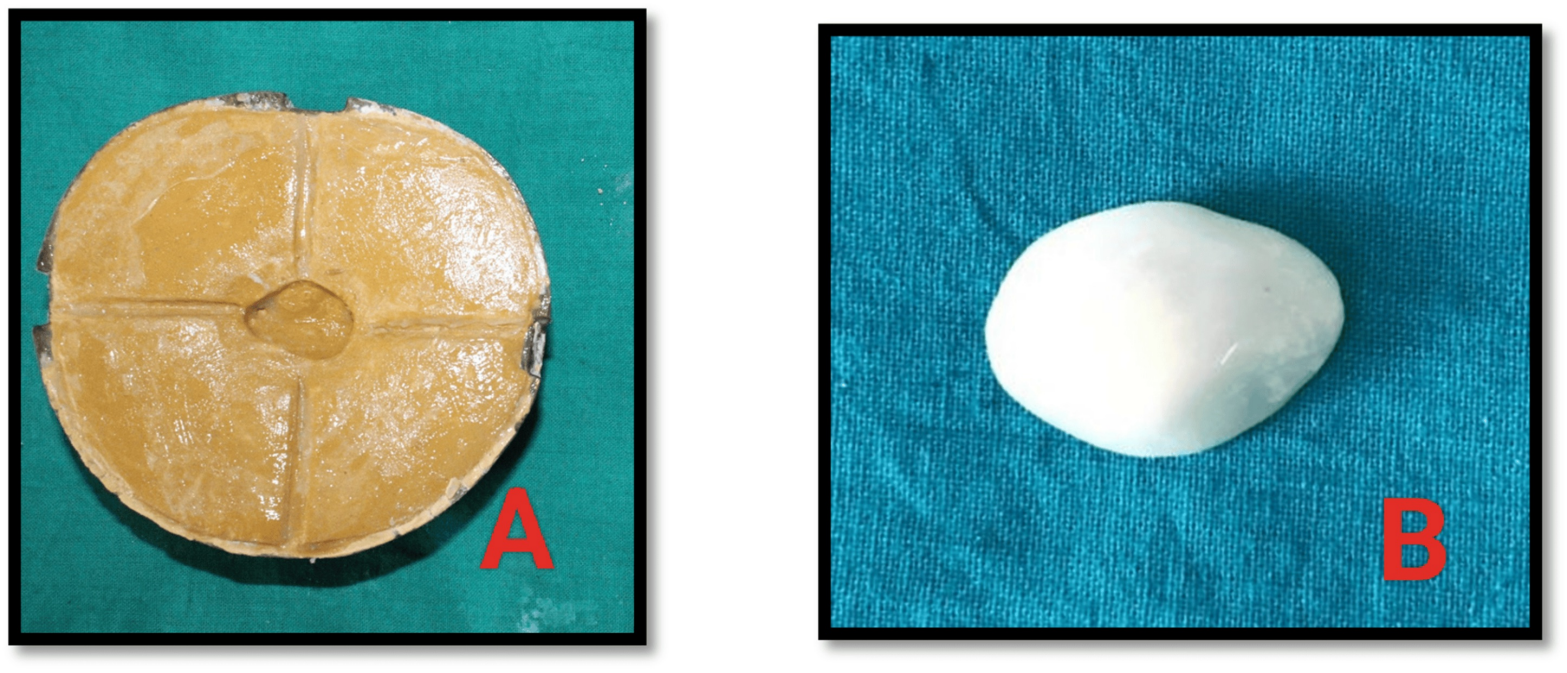
A: Dewaxed mold. B: Scleral part of prosthesis

**Figure 7:**
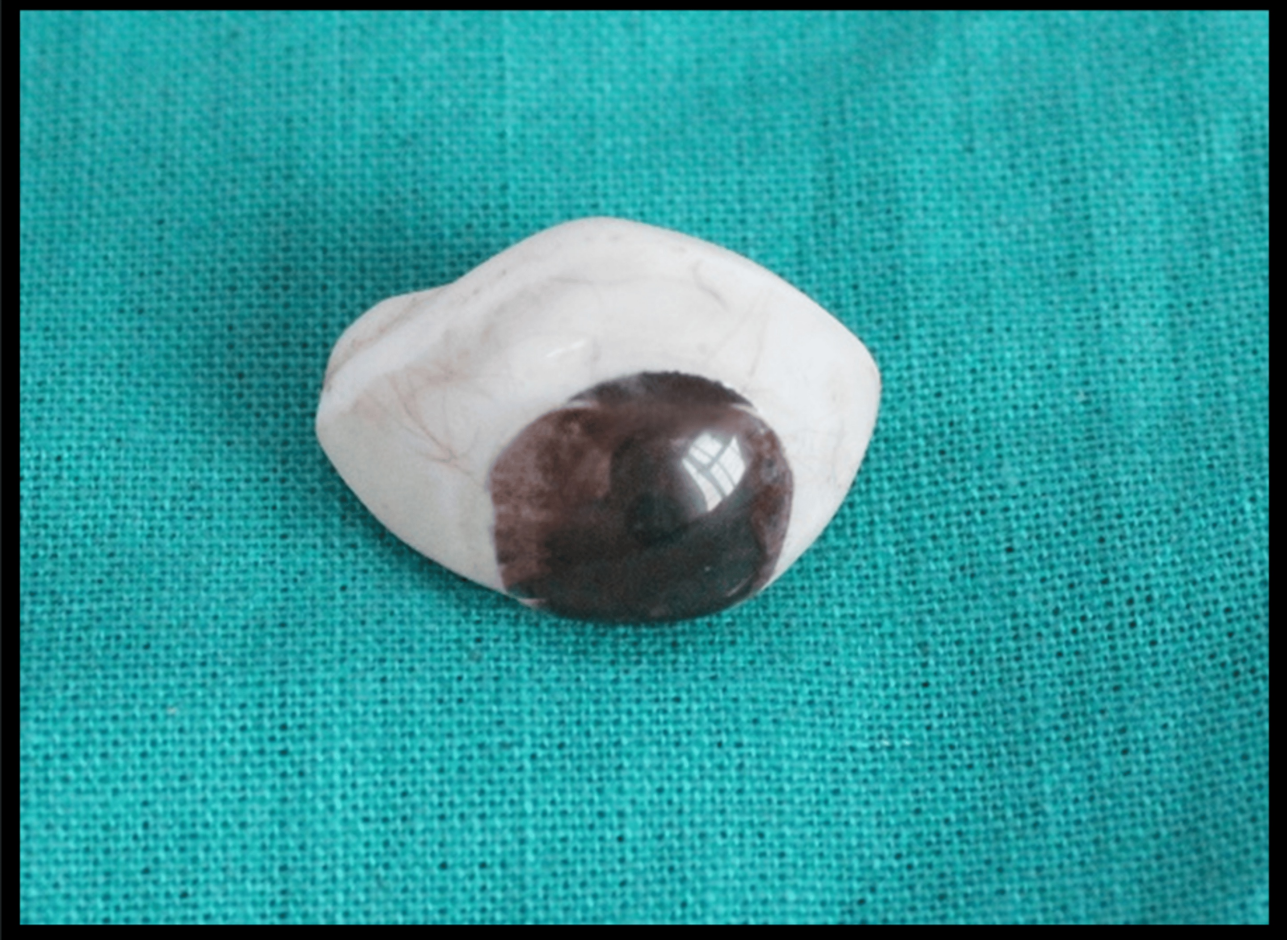
Final prosthesis

**Figure 8:**
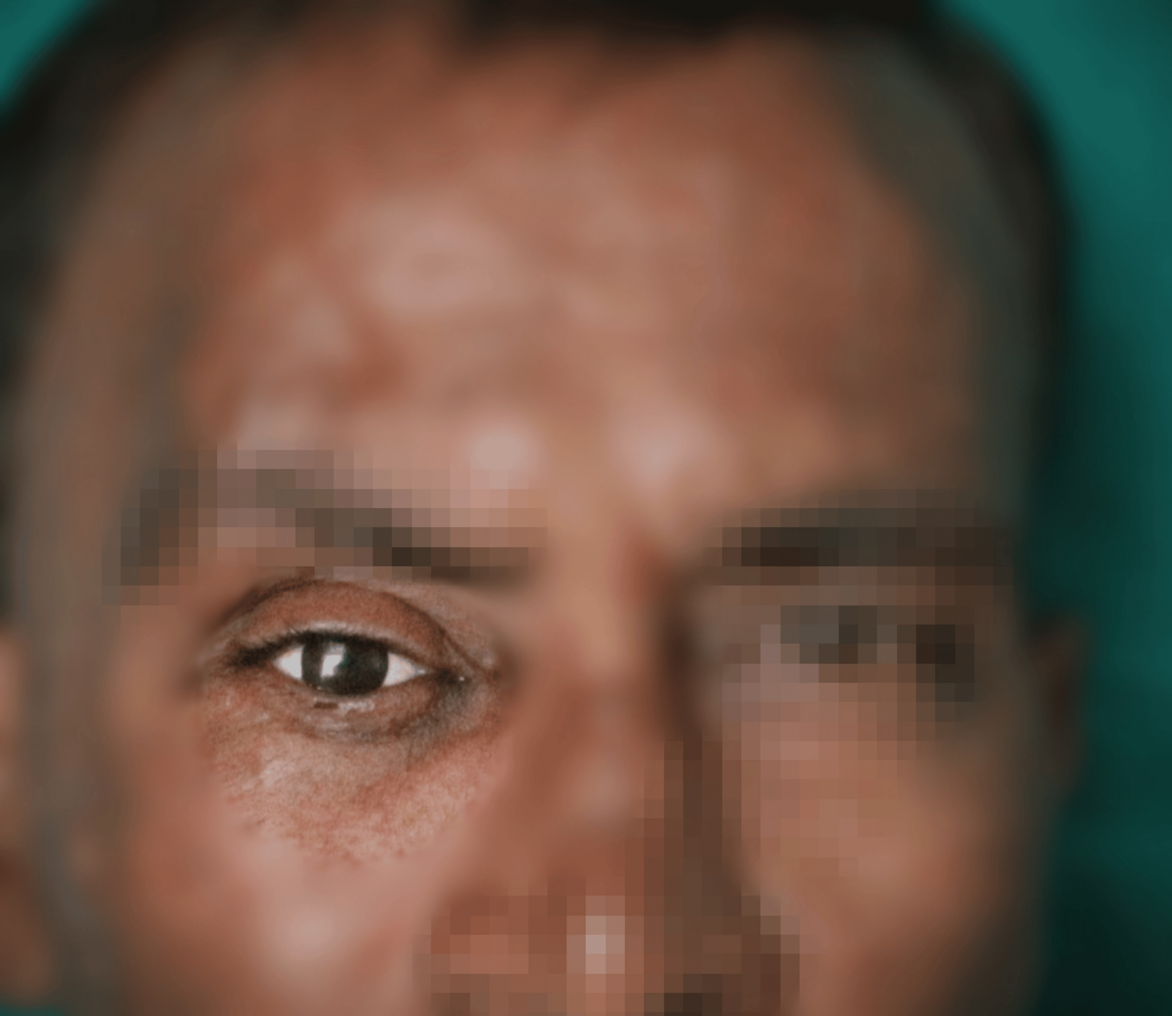
Final prosthesis insertion

Petroleum jelly application was done around the ocular area. Preliminary impression was made by injecting regular body (Reprosil, Dentsply Sirona, York, PA, USA) in the intraocular space and supported by putty consistency polyvinyl siloxane impression material (Aquasil, Dentsply Sirona, York, PA, USA) (Figure 2). Preliminary cast was poured in Type III dental stone (Kalabhai Karson Pvt. Ltd., Mumbai, India). This was followed by an acrylic custom tray fabrication (Cold Cure Acrylic Resin, Dental Products of India (DPI), Mumbai, India) with a handle made of a syringe cap (Figure 3). Final impression was made in light body consistency polyvinyl siloxane impression material (Photosil, Dental Products of India (DPI), Mumbai, India). The light body was injected using a cartridge, and the patient was asked to do upward, downward, and sideward movements to accurately record the intraocular space (Figure 4). Master cast was poured using the split cast technique in Type IV dental stone (Kalabhai Karson Pvt. Ltd., Mumbai, India). A wax pattern try-in was done, and the patient was asked to perform up, down, and side movements to check the retention of the wax pattern (Figure 5A). Markings were done on the patient’s face to accurately ascertain the position of the iris. Color match was done using the stock eye (Figure 5B). Flasking of the wax pattern was done, and a heat-cure tooth molding powder (Heat Cure Acrylic Resin, Dental Products of India (DPI), Mumbai, India) was used to fabricate the scleral part of the prosthesis (Figure 6). After finishing and polishing of the scleral shell, the iris was hand-painted to match the color of the contralateral eye, and fibers were incorporated in the shell to mimic the arteries for the characterization of the prosthesis (Technovent Ltd., Bridgend, UK). A thin layer of clear acrylic resin was placed on the prosthesis and cured (Heat Cure Acrylic Resin, Dental Products of India (DPI), Mumbai, India) (Figure 7). At the time of insertion, fit and retention of the prosthesis were checked (Figure 8). The patient was given instructions on the insertion and removal of the prosthesis along with hygiene considerations.

The patient was reviewed at one, three, and six months. The prosthesis remained well retained, with no reported complaints, and the patient was satisfied with its esthetics and overall quality.

## Discussion

Ocular defects are frequently reported in a prosthodontist’s clinic, which may be congenital or acquired. The rehabilitation of such defects is done with an ocular prosthesis. They have a long history of successful use, and the techniques and materials used in their fabrication have evolved over time [4]. The prosthesis can be custom-made for each patient or selected from a stock eye. The fabrication of custom-made ocular prostheses varies, particularly in iris disc production and impression techniques. More recent techniques use digital photographs of the contralateral eye with a grid method to replicate the iris [5,6]. Despite these advances, stock eyes remain widely used [7]. In some patients, however, stock eyes may not achieve satisfactory esthetic results because they adapt poorly to the socket contour, as in the present case [4]. Poor adaptation to the tissue bed may also cause tear pooling beneath the prosthesis, resulting in discomfort [8]. By contrast, custom prostheses adapt more closely to the tissue bed, helping to condition the tissues, reduce mucus secretion, and improve patient acceptance. They also provide better functional and esthetic outcomes than stock prostheses. In addition, customization allows more precise matching of iris size and color with the contralateral eye, further improving the esthetic result [6,8].

Various studies have found that custom-made ocular prostheses provide better aesthetic and functional outcomes compared to stock ocular prostheses [9,10].

Various digital approaches for ocular defect rehabilitation have been described in the literature. These methods involve acquiring patient data through CT scans, digitally designing the prosthesis, and fabricating it by 3D printing. However, 3D-printed resins lack adequate clarity, and the digital design process requires expertise and is not cost-effective [11].

Recent research has focused on developing biomimetic prosthetic systems. Their ability to respond organically to varying light intensities may further advance the development of biomimetic ocular prostheses [12].

This case report describes a simple and rapid method for fabricating a custom ocular prosthesis that produced a pleasing aesthetic outcome with good patient acceptance and satisfaction. The technique used chairside materials, making it practical for wider use by clinicians without the need for additional equipment or manpower.

## Conclusions

Custom-made ocular prostheses generally provide better esthetics and retention than stock prostheses. Their close adaptation improves patient comfort and confidence, while helping maintain a natural appearance and proper orientation during eye movements. Although other approaches may produce acceptable results, a customized prosthesis often leads to greater patient acceptance. It is also a cost-effective option, which supports its broader use in clinical practice. Because ocular injuries vary widely and each patient’s needs are unique, some individuals benefit more from a custom-made prosthesis. Although fabrication may take longer and require some trial and adjustment, the improved esthetic and functional results justify the additional effort.
